# Characterization of the species *Malassezia pachydermatis* and re-evaluation of its lipid dependence using a synthetic agar medium

**DOI:** 10.1371/journal.pone.0179148

**Published:** 2017-06-06

**Authors:** Laura Puig, M. Rosa Bragulat, Gemma Castellá, F. Javier Cabañes

**Affiliations:** Veterinary Mycology Group, Department of Animal Health and Anatomy, Universitat Autònoma de Barcelona, Bellaterra, Catalonia, Spain; Agency for Science Technology and Research, SINGAPORE

## Abstract

The genus *Malassezia* includes lipophilic yeasts, which are part of the skin microbiota of various mammals and birds. Unlike the rest of *Malassezia* species, *M*. *pachydermatis* is described as non-lipid-dependent, as it is able to grow on Sabouraud glucose agar (SGA) without lipid supplementation. In this study we have examined the phenotypic variability within *M*. *pachydermatis* and confirmed its lipid-dependent nature using a synthetic agar medium. We used a selection of representative non-lipid-dependent strains from different animal species and three atypical lipid-dependent strains of this species, which were not able to grow after multiple passages on SGA. More than 400 lipid-dependent *Malassezia* isolates from animals were studied in order to detect the three lipid-dependent strains of *M*. *pachydermatis*. The identity of the atypical strains was confirmed by DNA sequencing. On the other hand, we have modified the Tween diffusion test, which is widely used in the characterization of these yeasts, by using a synthetic agar-based medium instead of SGA. This modification has proved to be useful for differentiation of *M*. *pachydermatis* strains, providing reproducible results and a straightforward interpretation. The finding of these peculiar lipid-dependent strains exemplifies the large variability within the species *M*. *pachydermatis*, which involves rare atypical strains with particular growth requirements.

## Introduction

The genus *Malassezia* includes lipophilic yeasts, which are part of the skin microbiota of various mammals and birds. Currently, the genus includes 17 species [1, 2], three of which have been recently proposed [3, 4]. Of all these species, *M*. *pachydermatis* is a zoophilic yeast frequently isolated from the skin of wild and domestic carnivores. Although *M*. *pachydermatis* is part of the normal microbiota of the skin and ear canal of these animals, under some predisposing factors it can overgrow and lead to the development of dermatitis and otitis. These diseases are common in dogs, and occur less frequently in other animals [5].

Unlike the rest of *Malassezia* species, *M*. *pachydermatis* is described as non-lipid dependent, as it is able to grow on Sabouraud glucose agar (SGA) without lipid supplementation. The remaining species of the genus (e.g. *Malassezia furfur*), require fatty acid supplementation for growth in culture, and consequently they are named lipid-dependent species. Complex culture media, such as modified Dixon agar (mDA) and Leeming and Notman agar provide a variety of fatty acids, required by these fastidious lipid-dependent species [2]. Although *M*. *pachydermatis* is the less lipid-demanding species of the genus, it has been shown that it requires the peptone components of SGA, which are highly complex and undefined, but provide fatty acids essential for this species [1].

However, some *M*. *pachydermatis* isolates from dogs have shown some inconsistent lipid dependence [6]. They were reported as markedly lipid-dependent isolates. Some of these isolates grew poorly when sub-cultured onto SGA. Nevertheless, most of them were able to form colonies typical of this species on SGA after some subsequent transfers on this medium. On the other hand, the isolation of *M*. *pachydermatis* strains unable to grow on SGA has been rarely reported [7–9].

More recently, the use of massive sequencing methods has allowed a deeper understanding of the genome of these yeasts. For instance, a typical fungal fatty acid synthase was not detected in the genome of the neotype strain of *M*. *pachydermatis* [10]. More interestingly, it has been also proved that the gene encoding for the fatty acid synthase is missing in the genomes of all *Malassezia* species [11]. Furthermore, these authors also mentioned that two *M*. *pachydermatis* strains were only able to grow with lipid supplementation in the synthetic yeast nitrogen base broth, confirming the unique lipid-dependent nature of all *Malassezia* species.

Different standard physiological tests used in the identification of yeasts have been proposed in order to characterize phenotypically *M*. *pachydermatis* (e.g. assimilation of carbon compounds, fermentation of carbohydrates) [12, 13]. However, due to their essential requirements for lipids standard assimilation tests are not applicable to these yeasts [14]. Nowadays, the physiological characterization of *M*. *pachydermatis* is based mainly on the evaluation of its ability to grow on SGA and on its ability to use certain polyoxyethylene sorbitan esters (Tweens 20, 40, 60 and 80) and Cremophor EL using a glucose/peptone agar-based medium (SGA) [1, 15].

The aim of the present study was to examine the phenotypic variability within the species *M*. *pachydermatis* and to confirm its lipid-dependent nature using a synthetic agar medium. To do this, SGA medium used in the Tween diffusion technique [1, 15] was replaced by a synthetic agar-based medium. Moreover, in this study, we have included three atypical lipid-dependent *M*. *pachydermatis* strains and confirmed their identity by DNA sequencing.

## Materials and methods

### Strains

A total of 19 strains of *M*. *pachydermatis* were studied. These were selected from our collection in order to obtain representative strains from different animal species with different health status and genetic types (**Table 1**). Swabs from the skin and the external ear canals of various animals were obtained for microbiologic examination. All samples were inoculated onto SGA and mDA with 0.05% of chloramphenicol and 0.05% of cycloheximide. These strains were obtained during routine veterinary procedures and with the verbal owner consent. Most of the strains selected for this study had been recovered from dogs, where *M*. *pachydermatis* is most frequently isolated, but we also included strains from animals where this species is more rarely isolated. Three atypical lipid-dependent strains of this species (MA-366, MA-374 and MA-380) were also included. More than 400 lipid-dependent *Malassezia* isolates from animals were studied in order to detect the three lipid-dependent strains of *M*. *pachydermatis*. The identity of all strains was confirmed by DNA sequencing. The neotype strain of *M*. *pachydermatis* CBS 1879 was also included. Strains were stored at -80°C [16].

**Table 1:** *Malassezia pachydermatis* studied, including original animal host, pathology, and LSU rRNA, ITS rRNA, CHS2 and beta-tubulin genotypes.

Strain	Host	Location	Pathology	LSU /ITS/CHS2/beta-tubulin genotypes ^a^
CBS 1879	Dog-9	Ear	Otitis	I/I/I/I
CBS 1884	Dog-10	Ear	Otitis	I/I/I/II
CBS 6535	Dog	Ear	Healthy	I/I/II/I
MA-13	Dog-1	Ear	Healthy	I/I/III/I
MA-52	Dog-2	Ear	Healthy	I/II/I/II
MA-56	Dog-2	Ear	Healthy	I/II/I/II
MA-94	Horse	Skin	Healthy	I/III/I/I
MA-107	Goat	Ear	Healthy	II/IV/IV/III
MA-140	Cat-1	Ear	Healthy	I/V/V/IV
MA-195	Dog-3	Ear	Otitis	I/VI/I/I
MA-280	Dog-4	Ear	Otitis	III/VII/IV/III
MA-312	Cat-2	Ear	Otitis	IV/VIII/VI/V
MA-356	Dog-5	Ear	Otitis	III/IV/VII/VI
MA-366 ^c^	Dog-6	Ear	Healthy	V/XII/IX/VIII ^b^
MA-374 ^c^	Cow	Ear	Healthy	V/XII/IX/VIII ^b^
MA-380 ^c^	Dog-7	Ear	Healthy	III/XIII/IV/IX ^b^
MA-475	Pig	Ear	Healthy	II/IX/VIII/VII
MA-579	Cat-3	Skin	Dermatitis	IV/X/V/IV
MA-1382	Dog-8	Ear	Otitis	V/XI/IX/VIII

^a^ Genotypes determined in a previous sequencing study [18].

^b^ Genotypes determined in the present study.

^c^ Lipid-dependent strains.

Naming source: CBS, Centraalbureau voor Schimmelcultures; MA, culture collection of the Veterinary Mycology group.

### Morphological and physiological characterization

*M*. *pachydermatis* strains were streaked on mDA (36 g of malt extract (Oxoid S.A., Madrid, Spain), 10 g of bacteriological peptone (Oxoid S.A., Madrid, Spain), 20 g of desiccated ox bile (Sigma-Aldrich S.L., Madrid, Spain), 15 g of agar bacteriological (Oxoid S.A., Madrid, Spain), 10 ml of Tween 40 (Sigma-Aldrich S.L., Madrid, Spain), 2 ml of glycerol (Sigma-Aldrich S.L., Madrid, Spain) and 2 g of oleic acid (MP Biomedicals LLC., Illkrich, France) per liter; pH 6.0) [1] and incubated at 32°C. When fully developed colonies were observed (after 3–4 days of incubation), these were streaked on SGA (Oxoid S.A., Madrid, Spain) and incubated at 32°C. Strains that did not grow on SGA after 4 days were repeatedly inoculated, up to five times, to confirm their lipid dependence [6]. Morphological characteristics were observed after 7 days of incubation at 32°C on mDA. Physiological characterization was based on the splitting of esculin due to beta-glucosidase activity, catalase reaction and growth at 37°C, 40°C, 42°C and 45°C on mDA [1].

The ability to assimilate Tween 20 (MP Biomedicals LLC., Illkrich, France), Tween 40, Tween 60 (Merk KGaA, Madrid, Spain), Tween 80 (MP Biomedicals LLC., Illkrich, France) and Cremophor EL (Sigma-Aldrich S.L., Madrid, Spain) was tested with the Tween diffusion test on SGA [1]. On the other hand, the Tween diffusion test was also performed on yeast nitrogen base agar (YNBA). The composition of the medium was 6.7 g yeast nitrogen base (BD Difco S.A., Madrid, Spain), 20g agar bacteriological per liter (pH 5.4). For each strain, 18 ml of YNBA were melted and allowed to cool to about 50°C. Three ml of a yeast suspension were added to the medium. The suspension was obtained by inoculating two loopfuls of growing yeast in 3 ml of sterile distilled water. The agar mixture was poured onto a petri dish and when the medium was solidified, five wells of 2 mm in diameter were punched on the surface and filled with 15 microlitres of Tween 20, 40, 60, 80 and Cremophor EL, respectively. Plates were incubated for 10 days at 32°C, and growth was checked every 24 hours. Glucose assimilation as a unique carbon source was tested using also YNBA, following the same technique. When the medium was solidified, three equidistant wells of 2 mm in diameter were punched on the agar. Afterwards, each well was filled with 15 microlitres of a glucose (VWR International Eurolab S.L., Barcelona, Spain) dilution in sterile distilled water at different concentrations (1%, 2% and 4%). Plates were incubated at 32°C for 10 days and growth was checked every 24 hours. A *Rhodotorula glutinis* strain (RH-2) from our collection was used as control. All tests were performed by duplicate.

All strains were also streaked on YNBA supplemented with 10 g/1000 ml peptone and 40 g/1000 ml glucose, on YNBA with 10 g/1000 ml peptone, on YNBA with 40 g/1000 ml glucose and on YNBA with palmitic acid (MP Biomedicals LLC., Illkrich, France) at different concentrations (12 g/1000 ml, 6 g/1000 ml, 0.6 g/1000 ml and 0.06 g/1000 ml).

### DNA extraction, amplification, sequencing and phylogenetic analyses

DNA was extracted from 4-day old cultures on mDA of strains MA-366, MA-374 and MA-380, according to the FastDNA Spin kit protocol with the FastPrep FP-24 instrument (MP Biomedicals, Biolink, Barcelona, Spain). DNA was stored at -20°C until used as a template for PCR. Internal transcribed spacer (ITS) region (including the genes ITS1, 5.8S rRNA and ITS2), large subunit of the ribosomal RNA (LSU rRNA) region, chitin synthase 2 (CHS2) and beta-tubulin genes were amplified and sequenced, using the primers and the protocols described previously [17]. Sequences of the four genes of the remaining strains had been characterized previously [18].

For the phylogenetic analyses, LSU rRNA sequences of *M*. *pachydermatis* strains studied were aligned using Clustal X v2.0.12 [19], and regions of ambiguous alignment were removed with Gblocks [20]. A maximum likelihood analysis was conducted using MEGA 6 software [21] with 1,000 bootstrap replicates. A phylogenetic tree was constructed using the maximum likelihood method based on the Kimura 2-parameter model. The initial tree for heuristic search was obtained by applying the Neighbor-Joining method to a matrix of pairwise distances estimated using the Maximum Composite Likelihood (MCL) approach. The rate variation model allowed for some sites to be evolutionarily invariable. Clades that were supported by bootstrap values (bs) of ≥70% were regarded as strongly supported. Sequences of *M*. *furfur* CBS 1878 and CBS 7019, *Ustilago maydis* ATCC MYA-4924 and *Cryptococcus neoformans* CBS 132 were selected as outgroup for the tree construction.

## Results

### Morphology and physiology

Differential phenotypic characteristics of the studied strains are summarized in **Table 2**. All strains were able to grow on mDA and on SGA at 32°C, except for the strains MA-366, MA-374 and MA-380, which were unable to grow on SGA, confirming their lipid dependence. The microscopic examination of the lipid-dependent strains showed ellipsoidal yeast cells with buds formed on a broad base, characteristic of *M*. *pachydermatis*.

**Table 2:** Main differential phenotypic characteristics of the studied *M*. *pachydermatis* strains.

					Tween diffusion test									
Strain	Growth on SGA	Growth on mDA	Growth at 42°C	beta–glucosidase activity	with SGA					with YNBA				
					T 20	T 40	T 60	T 80	CrEL	T 20	T 40	T 60	T 80	CrEL
CBS 1879	+	+^1^	w	-	+	+	+	+	+	+^a^	+	+	+^a^	-
CBS 1884	+	+^1^	w	-	+^b^	+	+	+	+	+^a^	+	+	-	-
CBS 6535	+	+^1^	w	-	+	+	+	+	+	+^b^	+	+	+^a^	-
MA-13	+	+^1^	w	-	v	+^b^	+	+	+^b^	+^b^	+	+	w	w
MA-52	+	+^1^	w	-	-	+	+	+	+^b^	+^a^	+	+	w	w
MA-56	+	+^1^	+	-	-	+	+	+	+^b^	w	+	+	+^a^	+^a^
MA-94	+	+^1^	+	-	-	+	+	+	+^b^	w	+	+	+^a^	-
MA-107	+	+^2^	-	-	+	+	+	+	+^b^	w	+	+	+^a^	w
MA-140	+	+^1^	+	-	-	+^b^	+	+	+^b^	+^b^	+	+	+^a^	+^a^
MA-195	+	+^1^	+	-	v	+^b^	+	+	+^b^	+^a^	+	+	w	w
MA-280	+	+^2^	w	-	+	+	+	+	+^b^	+^b^	+	+	w	-
MA-312	+	+^1^	w	-	+^b^	+	+	+	+	+^b^	+	+	+	-
MA-356	+	+^1^	w	-	v	+^b^	+	+	+^b^	+	+	+	+^a^	-
MA-366	-	+^1^	-	+	+^a^	+	+	+	w	+	+	+	+	w
MA-374	-	+^1^	-	+	+^a^	+	+	+	w	+	+	+	+	w
MA-380	-	+^1^	-	+	+^a^	+	+	+	w	+	+	+	+	w
MA-475	+	+^1^	+	-	-	+^b^	+	+	+^b^	+^b^	+^b^	+	+^a^	-
MA-579	+	+^2^	-	-	w	+	+	+	+	+^a^	+	+	w	+^a^
MA-1382	+	+^1^	w	-	w	+^b^	+	+	+^b^	+	+	+	-	-

Growth on SGA (Sabouraud Glucose Agar) at 32°C; Growth on mDA (modified Dixon Agar) after 7 days of incubation at 32°C: +^1^ colony diameter of 2–5 mm

+^2^ colony diameter of <1 mm; Growth at 42°C on mDA after 7 days of incubation.

Tween diffusion test [1, 15] with SGA and YNBA (Yeast nitrogen base agar): +, good growth; w, weak growth; +^a^, growth at a distance of the well where the substrate was placed; +^b^, ring of growth inhibition at a distance of the well; -, growth inhibition; v, variable results between replicates.

Naming source: CBS, Centraalbureau voor Schimmelcultures; MA, culture collection of the Veterinary Mycology group.

After 7 days of incubation on mDA at 32°C two types of *M*. *pachydermatis* colonies were observed. Most of the strains formed colonies that were 2–5 mm in diameter, while strains MA-107, MA-280 and MA-579 formed colonies <1 mm in diameter. All strains were also able to grow on mDA at 37°C and 40°C, while at 42°C growth of strains MA-107, MA-366, MA-374, MA-380 and MA-579 was inhibited. All strains failed to grow at 45°C. None of the strains showed beta-glucosidase activity, except for the three lipid-dependent strains. The catalase reaction was positive for all strains.

**Fig 1:**
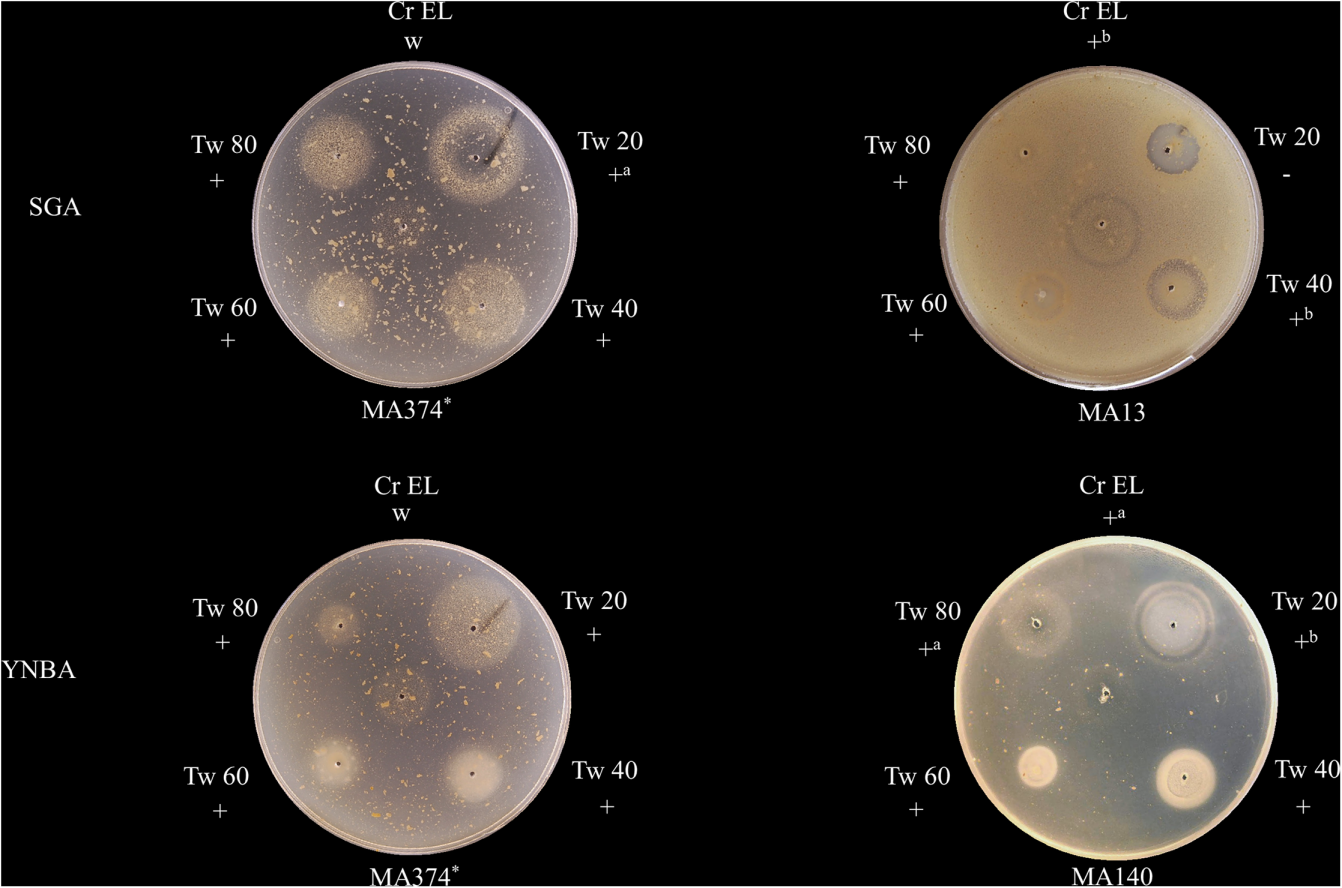
Growth patterns of *M*. *pachydermatis* strains in the Tween diffusion test with SGA and YNBA, after 7 days of incubation at 32°C. The growth patterns were defined as good growth (+); weak growth (w); growth at a distance of the well where the substrate was placed (+^a^); ring of growth inhibition at a distance of the well (+^b^); growth inhibition (-); *: lipid-dependent strain.

The Tween diffusion test was performed on SGA and on the synthetic medium without lipids YNBA. Using SGA, all strains except for MA-366, MA-374 and MA-380 grew on the entire surface of the agar, while on YNBA growth was only observed around the lipid supplements. On both media, although some intermediate growth patterns were found, five main assimilation patterns were observed around the lipid supplements (**Fig 1**). These patterns were defined as good growth (+), weak growth (w), growth at a distance of the well where the substrate was placed (+^a^), ring of growth inhibition at a distance of the well (+^b^), and growth inhibition (-). Using YNBA, growth profiles between replicates were identical. However, variable growth patterns were observed in some strains with Tween 20 on SGA.

**Fig 2:**
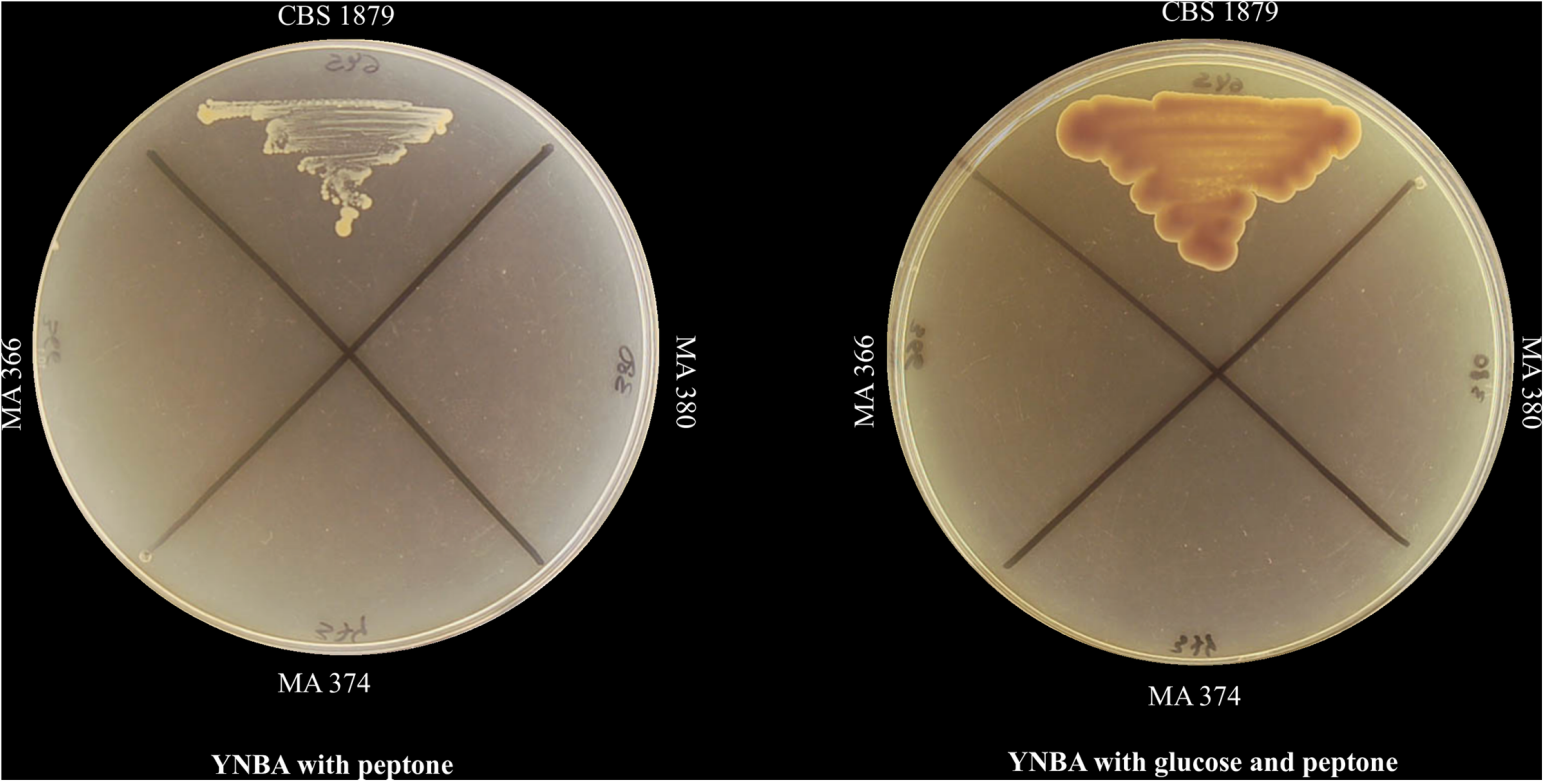
Growth of the neotype strain of *M*. *pachydermatis* (CBS 1879) on YNBA supplemented with peptone (10 g/1000 ml) and on YNBA supplemented with glucose (40 g/1000 ml) and peptone (10 g/1000 ml). None of the lipid-dependent strains (MA-366, MA-374 and MA-380) was able to grow on this media.

None of *M*. *pachydermatis* strains was able to assimilate glucose as a unique carbon source on YNBA after 10 days of incubation. *Rhodotorula glutinis* RH-2 used as control presented good growth at 2% and 4% glucose concentrations. Glucose assimilation profiles were identical between replicates. All strains were able to grow both on YNBA supplemented with peptone and glucose, and on YNBA with peptone only at 32°C, except for the lipid-dependent strains MA-366, MA-374 and MA-380 (**Fig 2**). The growth of the non-lipid-dependent strains was better on the peptone and glucose containing medium than on the medium containing only peptone. None of the strains grew on YNBA supplemented with palmitic acid at the different concentrations tested.

### DNA sequencing and phylogenetic analysis

**Fig 3:**
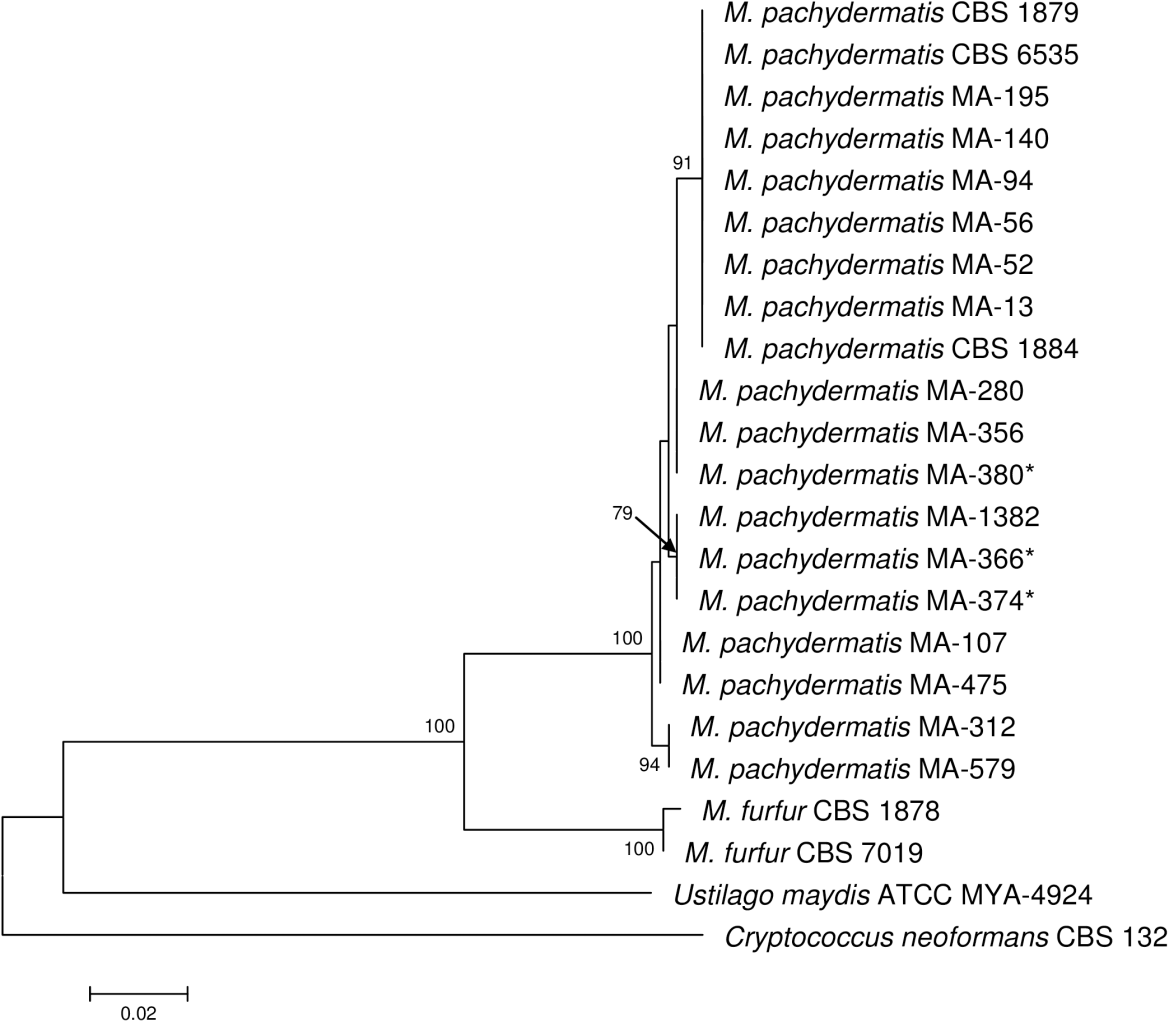
Molecular phylogenetic tree inferred from maximum likelihood analysis of LSU sequences of *Malassezia pachydermatis* strains. Bootstrap values > 70% in 1,000 replications are shown at the nodes. Sequences of *M*. *furfur* CBS 1878 and CBS 7019, *Ustilago maydis* ATCC MYA-4924 and *Cryptococcus neoformans* CBS 132 as outgroup were selected for the tree construction.* Lipid-dependent strains.

LSU rRNA region was successfully amplified for the three lipid-dependent strains (MA-366, MA-374 and MA-380), resulting in a product of 580 bp. A search on GenBank database using BLAST [22] revealed that the sequences of these strains had a percent identity of 99% to the sequence of the neotype strain of *M*. *pachydermatis* CBS 1879. Strains MA-366 and MA-374 showed identical LSU sequences and had an identity of 100% to *M*. *pachydermatis* MA-1382, while strain MA-380 had an identity of 100% to *M*. *pachydermatis* MA-280 (**Table 1**). The phylogenetic tree of LSU rRNA sequences revealed that *M*. *pachydermatis* strains formed a well-supported cluster, with 100% bootstrap support (**Fig 3**).

ITS rRNA, CHS2 and beta-tubulin genes were also amplified and sequenced for the lipid-dependent strains. Strains MA-366 and MA-374 showed identical ITS, CHS2 and beta-tubulin sequences. Length of the ITS region was 730 bp and constituted a new genotype (genotype XII), whose sequence has been deposited in GenBank under the accession number KY655274. Sequences of CHS2 (489 bp) and beta-tubulin (952 bp) genes matched previously described genotypes IX and VIII, respectively. Sequences of ITS (720 bp) ανd beta-tubulin (952 bp) of strain MA-380 constituted new genotypes namely genotype XIII (accession no. KY655275) and genotype IX (accession no. KY655276), respectively, whereas CHS2 sequence matched genotype IV previously described. The pairwise differences among sequences of the new genotypes from lipid-dependent strains and the previously described genotypes ranged from 0.1 to 7.0% and 0.1 to 3.4% for ITS and beta-tubulin genes, respectively. These genetic analyses confirmed the identification of the lipid-dependent strains as *M*. *pachydermatis*.

## Discussion

Guillot et al. [15] proposed the first practical approach for phenotypic characterization of *Malassezia* species. It is based mainly on the ability to utilize certain lipid compounds (e.g. Tweens) using a diffusion test on SGA. This method is still currently used for differentiation of *Malassezia* species [1]. Nonetheless, in some cases it may not be accurate enough to achieve a correct identification of atypical strains [7, 9, 23–25]. Therefore, some molecular methods (e.g. rDNA sequencing) are necessary to confirm the identification to species level of *Malassezia* yeasts [1, 17, 26].

In routine fungal identification, isolates of *M*. *pachydermatis* are usually identified by microscopic morphology and by its ability to grow on SGA. In the first steps of the identification scheme of *Malassezia* species, it is considered that if growth on SGA is observed, the yeast is *M*. *pachydermatis* [1, 15]. Although in most cases this assumption is correct, in our study three *M*. *pachydermatis* strains did not grow on SGA. Previous studies have reported the existence of atypical lipid-dependent *M*. *pachydermatis* isolates [7–9].

In routine *Malassezia* spp. identification, SGA is widely used. In *M*. *pachydermatis*, the term “non-lipid-dependent species” is clearly linked to the use of this medium and it means that this yeast is able to grow on SGA. Interestingly, most *M*. *pachydermatis* isolates grow on this medium. However, this term, used in a wide sense, would not be correct since recently it has been proved that the gene encoding for the fatty acid synthase is missing in the genomes of all *Malassezia* species [11] and atypical lipid-dependent *M*. *pachydermatis* isolates have been described in the present study. Nowadays, in the case of this species, we think that it would be more suitable to use the term “traditionally described as non-lipid dependent”.

In the present study, all strains were able to grow on mDA from 32°C to 40°C, and showed catalase activity, in agreement with previous studies [1]. Most of the strains were able to grow at 42°C, except for the three lipid-dependent strains and strains MA-107, isolated from a goat, and MA-579, recovered from a cat. Almost all the strains formed similar colonies of normal size and appearance at 32°C. However, a few strains showed small colonies at this temperature. Similarly, previous studies reported the presence of *M*. *pachydermatis* strains that grew poorly on SGA and had a smaller colony diameter [6, 27, 28]. All studied strains were unable to split esculin, except for the three lipid-dependent strains. In this species, variable test results for beta-glucosidase activity have been reported [1].

In our study, the Tween diffusion test was performed on SGA and on YNBA. On SGA, all isolates except for the lipid-dependent strains were able to grow on the entire surface of the agar. All strains were able to assimilate Tweens 40, 60 and 80 and Cremophor EL, showing distinct growth patterns. Most of the strains also assimilated Tween 20. However, some strains showed different assimilation patterns using this lipid source. Following this technique, different assimilation patterns have been reported for *M*. *pachydermatis* strains [1]. For instance, growth inhibition around the four Tweens was reported by Guillot et al. [15], while other strains have been reported to assimilate Tweens 40, 60 and 80 [13]. We observed a particular assimilation pattern on both culture media, corresponding to a ring of growth inhibition at a distance of the well where the substrate was placed (+^b^). Previously, a similar pattern on SGA had been described as secondary or delayed growth, after the diffusion of the lipid supplements through the medium [1]. Nonetheless, in our study this pattern was observed within the first 24 hours of incubation. Therefore, this pattern could be due to the interaction of lipid supplements and components of the medium, but in depth studies should be performed in order to confirm this hypothesis.

We think, that the Tween diffusion test could be improved substituting SGA for YNBA. The lack of intra- and interlaboratory reproducibility of this technique are due, in part, to the peptone components of SGA, which are highly complex and undefined, and may vary from batch to batch [29]. In our study, using YNBA, the assimilation patterns were identical between replicates, showing a higher reproducibility. On the other hand, growth was only observed around the lipid supplements, facilitating the visualization of growth patterns. Moreover, more assimilation patterns were observed among strains, allowing the visualization of differences that could not be detected using SGA.

In the present study, all *M*. *pachydermatis* strains were unable to assimilate glucose as a sole source of carbon in YNBA, which confirmed that a minimum amount of lipid is required for *M*. *pachydermatis* growth in this medium. Recently, Wu et al [11], using genomic analyses, revealed that a larger set of genes involved in carbohydrate metabolism had been lost in *Malassezia* species, concordant with adaptation to skin’s carbohydrate-deficient environment. Moreover, these authors pointed out that, at the same time, a wide expansion of lipid hydrolases occurred in these yeasts.

All strains examined in our study, with the exception of the lipid-dependent strains, grew on YNBA with peptone. Therefore, some components of peptone promoted the growth of these yeasts. However, their growth was more abundant on YNBA containing glucose and peptone. Thus, glucose increased the growth of the non-lipid-dependent strains in these conditions. However, this was not the case for the atypical lipid-dependent strains of *M*. *pachydermatis*, which did not grow under any of these conditions tested. On the other hand, it has been reported that the peptone in SGA contains palmitic acid and lesser amounts of other fatty acids, and it has been suggested that those lipids are required for *M*. *pachydermatis* growth [11]. In our work, none of the strains was able to grow on YNBA supplemented with palmitic acid at various concentrations. Possibly, the other fatty acids contained in peptone, among other compounds, are required for the growth of these yeasts. In fact, Wu et al [11] showed that the number of lipases varies among the different *Malassezia* species. These authors hypothesized that the more lipases these species had, the more lipids they could use, and consequently, they could live in more diverse ecosystems. Due to the genetic diversity observed in *M*. *pachydermatis* strains [18, 30–32], we also hypothesize that the same could happen within this species.

In our study, the three lipid-dependent strains of *M*. *pachydermatis* showed some differential phenotypic characteristics. Besides their inability to grow on SGA, these strains showed beta-glucosidase activity and unique Tween assimilation profiles in SGA and YNBA. Sequencing of the ITS and LSU rRNA regions, beta-tubulin and CHS2 genes confirmed that the three lipid-dependent strains belonged to the species *M*. *pachydermatis*. LSU rRNA and CHS2 sequences from the lipid-dependent strains matched previously characterized *M*. *pachydermatis* genotypes and the new ITS and beta-tubulin genotypes from these lipid-dependent strains did not exceed the variation generally observed to occur in *M*. *pachydermatis* [18]. Besides, in the phylogenetic tree of the LSU rRNA sequences, the lipid-dependent strains were grouped and interspersed with the non-lipid dependent *M*. *pachydermatis* strains.

In this study we have demonstrated the significant intraspecific diversity within the species *M*. *pachydermatis*. On the other hand, we have modified the Tween diffusion test for *M*. *pachydermatis* study using the synthetic medium YNBA, which has proved to be useful for differentiation of *M*. *pachydermatis* strains, providing reproducible results and a straightforward interpretation. Further studies are needed to assess the usefulness of this modified technique to distinguish the species of *Malassezia*. Moreover, testing glucose assimilation in YNBA we demonstrated that *M*. *pachydermatis* requires a minimum amount of lipid for growth in culture, as those provided by the complex medium SGA. On the other hand, we have characterized three lipid-dependent *M*. *pachydermatis* strains isolated from domestic animals. The finding of these peculiar strains exemplifies the huge variability within *M*. *pachydermatis*, which involves atypical strains with particular growth requirements.
